# Consumer Attitudes toward Vertically Farmed Produce in Russia: A Study Using Ordered Logit and Co-Occurrence Network Analysis

**DOI:** 10.3390/foods10030638

**Published:** 2021-03-17

**Authors:** Yuki Yano, Tetsuya Nakamura, Satoshi Ishitsuka, Atsushi Maruyama

**Affiliations:** 1 Department of Food and Resource Economics, Chiba University, 648 Matsudo, Matsudo 271-8510, Chiba, Japan; a.maruyama@faculty.chiba-u.jp; 2 Department of International Business Management, Kyoei University, 4158 Uchimaki, Kasukabe 344-0051, Saitama, Japan; t-nakamura@kyoei.ac.jp; 3 Department of International Agriculture and Horticulture, Hirosaki University, 3 Bunkyo-cho, Hirosaki 036-8561, Aomori, Japan; s-ishi@hirosaki-u.ac.jp

**Keywords:** consumer attitude, novel food technology, vertical indoor farming, network analysis, word co-occurrence

## Abstract

Vertical indoor farming under artificial lighting has gained attention as a novel means of food production. However, consumer acceptance of vegetable crops grown under artificial conditions is not well understood. Our nationwide online survey of 289 Russians gathered attitudes and opinions toward vertically farmed vegetables. Employing an ordered logit model and a two-mode co-occurrence network analysis, we show how respondents’ attitudes relate to their key demographic characteristics and opinions about the vegetables. Results indicate that respondents’ attitudes are heterogeneous and related to their region of residence, income level, and opinions regarding nutrients, safety, and taste. Respondents in the Central and Volga districts exhibited less favorable attitudes. Less favorably inclined respondents viewed the produce as unnatural, less nutritious, bad-tasting, and even dangerous, presumably because of misconceptions or lack of knowledge. On the other hand, respondents with monthly income above RUB 60,001 (1018 USD, 867 EURO) had relatively positive attitudes toward such vegetables. Respondents having positive attitudes saw the vegetables as safe, tasty, and of good quality. We discuss the political and commercial implications of these findings.

## 1. Introduction

Vertical indoor farming using multi-layer growing systems under artificial lighting (e.g., plant factories or city farming) has attracted worldwide attention as an innovative food production technology [1]. By controlling temperature, humidity, light, water, carbon dioxide, and nutrient concentrations in man-made structures, vertical farms are used to create favorable year-round conditions for plant growth and to stably produce high-yielding, high-quality agricultural products nearly anywhere [2]. As a complement to traditional farming, vertical indoor farming increases food supply, local food security, and healthier diets [3] by offering fresh, nutritious/functional, pesticide-free produce in markets where it is rarely seen. In Russia, it could certainly mitigate winter vegetable shortages. It may also contribute to reduce the adverse impact of COVID-19 on food security [4] by enhancing local production for local consumption and minimizing human contact using automated growing systems and packaging bags.

Although vertical indoor farming offers benefits over traditional farming, its diffusion in most regions, including Russia, is in its early stages, owing to high investment and energy costs [5]. Extensive research is under way to improve the cost-performance of vertical farming. For example, energy-saving devices (e.g., light-emitting diode (LED) technologies) are being adopted [6]. Even if technical issues are overcome, the process and rationale of consumer acceptance of vertically farmed produce (mainly leafy vegetables) is not well understood [7]. Innovations, such as genetic modification, food irradiation, and nanotechnology, routinely confront consumer skepticism in today’s world, especially during the introductory phases [8,9,10,11,12,13,14]. Consumers inexplicably resist vegetables grown in vertical farms where the cultivation environment is artificially controlled. Thus, promoting the acceptance of this important farming system requires understanding consumers’ backgrounds and rationale for their attitudes before launching products into the market [13,15,16].

Previous studies showed that the acceptance of new food products or technologies varied across countries and was generally heterogenous among individuals [8,11,13,17,18,19,20]. These researchers have suggested that personal knowledge and awareness, trust in the food industry and sources of information, perceived “naturalness” of food production, ethical concerns, economic factors, and sociodemographic characteristics often affect consumers’ reactions to innovative food technologies. The phenomenon appears to be primarily affected by consumer perceptions of risks and benefits. Recent studies have investigated how food technology neophobia (i.e., fear of modern technologies) has affected attitudes toward technologically driven foods. Genetically modified and nano-foods are key examples [10,20,21,22]. These studies found that food technology neophobia, as measured by the Food Technology Neophobia Scale [23], reflected consumers’ risk/benefit perceptions, perceptions of naturalness, trust in media and other sources of information, and interest in new technologies. Thus, these metrics can be used to substantially predict consumer acceptance of new technology.

Few attempts have been made to investigate consumers’ opinions, attitudes, and expressed acceptance of vertically farmed produce. Kurihara et al. [24] revealed that many Japanese consumers thought vertically farmed vegetables were sanitary, safe, and/or fresh. However, one-fourth of surveyed respondents viewed them as artificial or low in nutritional value. One-fifth believed that they were not tasty. Coyle and Ellison [7] surveyed U.S. consumers’ perceptions of safety, quality, and naturalness of lettuce produced by vertical farming, greenhouse farming, and field farming. Respondents perceived the vertically farmed lettuce as “less natural”. Thus, they were less likely to buy it before alternatives. Considering these findings, it is expected that consumers are heterogeneous in their attitudes and some express negative opinions about the quality and/or naturalness of vertically farmed produce. As in the case with other food technologies, key demographic characteristics, such as gender, age, region of residence, and income, are expected to be associated with these attitudes [8,11]. To date, however, no study has applied an exploratory approach to identify consumers’ opinions about such produce, nor have they examined how consumers’ opinions and characteristics are linked to their attitudes.

Marketing and consumer research routinely use free-association techniques to gauge consumer opinions, perceptions, ideas, and purchase decisions [25,26,27,28,29,30,31]. In these situations, researchers present stimulus words or phrases to interviewees and ask them to write down what comes to mind. They process and analyze the unstructured data using well-known text-mining techniques that enable them to uncover hidden knowledge or patterns from a large amount of information, which would otherwise be difficult and particularly time-consuming to analyze. Text mining involves converting unstructured data into structured forms and analyzing them using mining techniques (e.g., multi-dimensional scaling, correspondence analysis, and co-occurrence network analysis).

Among these techniques, co-occurrence network analysis provides a graphical visualization of the relationships between extracted words and has become increasingly popular, because it facilitates systematic discovery of primary themes and keywords in the texts. Because words are directly connected to one another or to specific categories, it is much easier to identify groups of words having similar appearance patterns using this method compared with the other methods mentioned [32]. Previous studies involved network analysis of one-mode data (i.e., one set of nodes) to study consumer opinions, perceptions, consciousness, or needs [31,33,34,35,36]. However, none have employed two-mode data (i.e., two sets of nodes). Use of two modes better reveals word groups that appear frequently as “the voice” of consumer groups having similar characteristics, attitudes, and behaviors.

The aim of this study was to reveal consumers’ attitudes and opinions toward vertically farmed leafy vegetables via a survey in Russia. Employing an ordered logit model and a two-mode co-occurrence network analysis, the study examined how consumer attitudes related to their key demographic characteristics and opinions about the vegetables. Our analysis should provide insight into the potential for consumer acceptance of such vegetables and the reasons for accepting or rejecting them. Thus, the results ought to have implications for policymakers, vertical farmers, and marketers.

## 2. Materials and Methods

### 2.1. Data Collection and Preparation

#### 2.1.1. Online Survey

We contracted SurveyMonkey^®^ (SurveyMonkey, San Mateo, CA, USA) for a nationwide online survey of Russian consumers, aged 20 and over, during November 2017. Among the 320 participants who responded to our questionnaire, 289 completed all questions. Table 1 shows that respondents were widely distributed geographically and by gender, age, family size, and monthly income. Few respondents were over age 60 or from the North Caucasus and Far East districts; more respondents were younger and from the Central and Northwest districts.

Participants answered the following open-ended question as a free-association task: “Recently, there are an increasing number of ‘vertical farms’ where leafy vegetables, such as lettuce, are grown under artificial lighting. What is your view or idea of ‘leafy vegetables grown under artificial lighting’? Please write as many ideas or thoughts as possible (at least three).” Following this task, respondents were asked to indicate their degree of favor toward vertically farmed vegetables using a five-point Likert scale: favorable = 5; somewhat favorable = 4; neutral = 3; somewhat unfavorable = 2; and unfavorable = 1.

#### 2.1.2. Procedures for Text Processing

**Figure 1:**
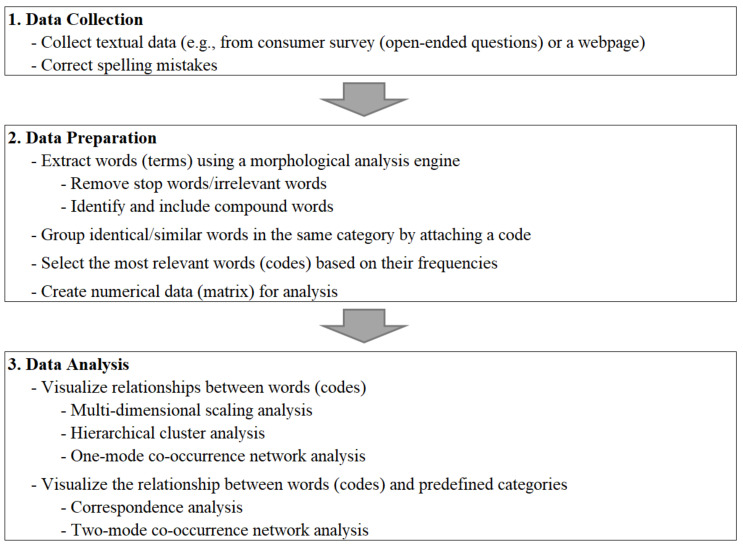
Text-mining procedure.

Figure 1 shows the flowchart of the text-mining process. Collected textual data from the first step were pre-processed using the free KH Coder text-mining software [32]. We pre-arranged for Russian text to be translated into English and for typographical errors to be repaired prior to text processing (e.g., “pprobably” was corrected to “probably”).

We extracted words from the text and calculated the frequency of each using Stanford’s Part-of-speech Tagger, an English morphological analysis engine available in KH Coder. Stopwords (e.g., “be,” “in,” “the,” “we”) were expunged at this stage. We extracted negating phrases (e.g., “not natural,” “don’t know,” “no vitamins”) as single words (e.g., “not_natural,” “not_know,” “no_vitamins”). We extracted adjectival comparisons (e.g., “less nutritious”) as single words (e.g., “less_nutritious”), and resituated adverbs (e.g., “not always safe”) after their adjectives (“not_safe always”).

From the list of extracted words, we grouped those having identical or similar meanings in the same category by attaching a code (i.e., a single word chosen from that category). For example, “not_natural,” “unnatural,” and “unnaturally” were merged and named (coded) “not_natural.” Similarly, “tasty” and “delicious” were grouped together and named “tasty.” Codes that appeared in less than 2% of all responses were eliminated because of low information content. Only the most relevant codes (hereafter simply “words”) were selected to create a numerical matrix for analysis. This makes the visualized results from data mining easier to interpret.

Data mining, including correspondence analysis and co-occurrence network analysis, can visualize relations between words and degrees to which respondents favored vertically farmed vegetables [27,37]. We used two-mode (i.e., bipartite) co-occurrence network analysis, because it can be used to systematically determine the most relevant words for each favorability option, as explained in Section 2.2.2.

### 2.2. Data Analysis

#### 2.2.1. Ordered Logit Model

To examine how key characteristics of respondents influence attitudes (favorability) toward vertically farmed vegetables, an ordered logistic regression was used. Let y_i_^*^ be an unobserved latent variable representing the favorability of the *i*th respondent. This is assumed to be a linear function of exploratory variables with a random-error term:

y_i_^*^ = β′x_i_ + ε_i_ (1)

where *β* is a *K* × 1 vector of parameters to be estimated, *x_i_* is a vector of exploratory variables showing characteristics of the *i*th respondent, and *ε_i_* is a mean-zero random-error term. Suppose the range of the latent variable is dissected by *J* − 1 thresholds into *J* regions (number of possible outcomes), thus *α*_0_ < *α*_1_ < ⋯ < *α_J_* denotes threshold parameters that determine the observed responses as follows:

*y_i_* = *j* ⇔ *α_j_*_−1_ < y_i_^*^ < *α_j_*, *j* = 1, 2, …, *J*, (2)

where *α*_0_ = −∞ and αJ = ∞. Using Equations (1) and (2) can be rewritten as

*y_i_* = *j* ⇔ *α_j_*_−1_ − *β*’*x_i_* < *ε_i_* < *α_j_* − *β*′*x_i_*, *j* = 1, 2, …, *J*, (3)

Assuming the error term is independently and identically logistically distributed, the probability that respondent *i* chooses outcome *j* is expressed as

*π_ij_* = *P*(*y_i_* = *j*|*x_i_*) = Λ(*α_j_* − *β*′*x_i_*) −Λ(*α_j_*_−1_ − *β*’*x_i_*), *j* = 1, 2, …, *J*, (4)

Λ(∙) denotes the cumulative distribution function of a logistic distribution. Maximum likelihood estimates of parameters can be obtained by maximizing this log-likelihood function:

log*L(β, α_1_, α_2_, …, α_j−1_; y, x) = Σ_i_Σ_j_ z_ij_*log *π_ij_* (5)

where *z_ij_* is an indicator variable that equals 1 if *y_i_* = *j* and 0 otherwise.

Our ordinal dependent variable, the degree of favorability, was coded as favorable = 5, somewhat favorable = 4, neutral = 3, somewhat unfavorable = 2, and unfavorable = 1. The independent variables included in the model were a gender dummy, dummies for age groups, dummies for region of residence, and dummies for income categories. We treated age and income as dummy variables because we used multiple-choice questions with different age cohorts and ranges of income in our survey to avoid asking direct questions about the respondent’s age and income.

A gender dummy was coded 1 for females and 0 for males. Using the age cohort of “20s” as the reference group, three dummy variables were created for age cohorts “30s,” “40s,” and “over 50” (coded 1 for respondents in the respective cohort and 0 otherwise). With respect to region of residence, dummy variables for each of the three federal districts, Central, Northwest, and Volga, where more than 10% of respondents lived in each, were created with other districts as the reference category (coded 1 for respondents living in the respective district and 0 otherwise). Finally, dummy variables for monthly income categories spanning RUB 20,001–40,000 (339–679 USD, 289–578 EURO using the monthly average exchange rate in November 2017 obtained from OECD.Stat: 1 USD = 58.92 RUB; 1 EURO = 69.21 RUB); RUB 40,001–60,000 (679–1018 USD, 578–867 EURO); RUB 60,001–80,000 (1018–1358 USD, 867–1156 EURO); and over RUB 80,001 (1358 USD, 1156 EURO) were created with “under RUB 20,000 (339 USD, 289 EURO)” as the reference category (coded 1 for respondents in the respective category and 0 otherwise). Stata 15.1 software performed all calculations (StataCorp, College Station, TX, USA).

#### 2.2.2. Co-Occurrence Network Analysis of Two-Mode Data

To reveal the relations between words extracted from participants’ text responses and their favorability toward vertically farmed vegetables, two-mode co-occurrence network analysis was used. This subsection provides an explanation while comparing the method with the one-mode co-occurrence network analysis method.

**Figure 2:**
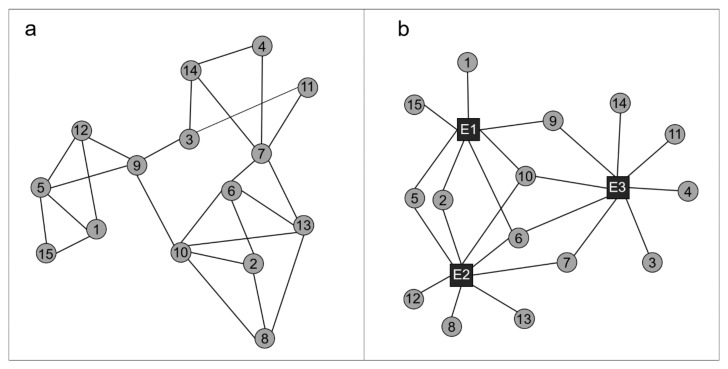
Examples of networks: (**a**) one-mode; (**b**) two-mode.

A network describes a set of objects (i.e., nodes) and the relations (i.e., edges) among them. The most common type of network is a one-mode network consisting of a set of nodes of the same type, including human relations or hyperlinks between websites. Figure 2a is an example of a one-mode network that illustrates the friendship relationships between 15 people in a hypothetical college class. Another important type of network is a two-mode network, which describes ties between two different sets of nodes [38]. For example, Figure 2b shows a two-mode network that describes the participation of 15 people (see the first set of nodes labeled with numbers) in three social events (see the second set of nodes labeled with E1, E2, and E3). Network analysis is a study approach that examines the structure of these types of networks based on graph theory.

Word co-occurrence network analysis combines network analysis with the conventional co-word analysis technique. It can be used to analyze the relationships between words extracted from texts. A one-mode co-occurrence network connects words (i.e., nodes) that appear often in the same context (i.e., sentence, paragraph, document) by their edges, using similarity measures (e.g., Jaccard and Cosine coefficients). Applying a community detection method to generated networks permits the systematic identification of word groups that reflect prominent themes from the text [31,33,36]. Compared with this, assuming that texts can be classified into categories beforehand, a two-mode co-occurrence network connects frequently appearing words within texts in a particular category to a node representing that category with edges based on similarity measures. Although co-occurrence network of two-mode data cannot be used to analyze the relationships between words, it is useful for identifying visually and systematically the most relevant words for each predefined category. Therefore, this method is suitable for our purpose.

The KH Coder can be used to create a map of a two-mode co-occurrence network that depicts words extracted from free-text answers to the above open-ended question as circular nodes and response options, indicating respondents’ favorability as square nodes, as with Figure 2b. An edge is placed between a word and option on the favorability scale (i.e., response option) if the word appears often in answers from the respondents who choose that option. For example, suppose “nitrate” appears often in the answers from the respondents choosing “unfavorable” option. Then, “nitrate” and “unfavorable” are connected by an edge.

We used the Jaccard coefficient [39] to measure the degree of co-occurrence between words and response options to reconsider the example above. Equation (6) calculates the Jaccard coefficient (i.e., degree of co-occurrence) between “nitrate” and “unfavorable:”

*J*(*nitrate, unf*) = |*nitrate* ∩ *unf*|/|*nitrate* ∪ *unf*| = *Nnitrate, unf*/(*Nnitrate* + *Nunf* − *Nnitrate, unf*) (6)

where 𝑁_𝑛𝑖𝑡𝑟𝑎𝑡𝑒_ denotes the number of respondents whose comments included “nitrate.” 𝑁_𝑢𝑛𝑓_ represents the number of respondents who chose the “unfavorable” response option. 𝑁_𝑛𝑖𝑡𝑟𝑎𝑡𝑒__,__𝑢𝑛𝑓_ represents the number of respondents whose comments included “nitrate” and those who chose the “unfavorable” response option.

Calculating the Jaccard coefficient for all combinations of words and response options generates a co-occurrence matrix from which a two-mode co-occurrence network map can be created. These are typically created using a limited number of edges in descending order of the Jaccard coefficient values. This structure allows us to identify the most relevant words for each response option.

## 3. Results

### 3.1. Favorability toward Vertically Farmed Leafy Vegetables

As shown in Table 2, 53.3% of respondents favorably or somewhat favorably viewed vertically farmed leafy vegetables. Of the respondents, 18.9% viewed them neutrally, and 27.8% somewhat unfavorably or unfavorably viewed them. Results indicate that Russian consumers have differing attitudes toward vertically farmed leafy vegetables, and they view their attributes differently.

### 3.2. Ordered Logistic Regression

To investigate the impact of respondents’ key characteristics on favorability, an ordered logistic regression was performed. Table 3 displays the estimation results for both the full model, which includes all variables, and the final model, which includes only variables selected via backward stepwise selection (i.e., statistically significant). The final model has a smaller Akaike information criterion (AIC), which indicates a better fit. The Chi-squared statistic (*χ*^2^_(4)_ = 14.7***) indicates that the model is statistically significant at 1%. However, the Pseudo *R*^2^ is only 0.02. Here, we interpret the estimated coefficients of the final model.

First, coefficients of regional dummies “Central” and “Volga” are negative and statistically significant at 5 and 10%, respectively. Respondents in those federal districts were less likely to be favorable toward vertically farmed leafy vegetables. The Northwest dummy was not significant, probably because the Central and Volga districts in western Russia are surrounded by semi-fertile agricultural areas and are relatively populous. They are also close to EU markets, and fresh leafy vegetables are more readily available. Respondents in those districts will probably find that vertically farmed vegetables are less necessary than respondents in districts surrounded by relatively unproductive land.

Second, dummies for high-income respondents (RUB 60,001–80,000, denoted “Income_68” and over RUB 80,001 denoted “Income_o8”) exerted significantly positive effects on the dependent variable. This indicates that higher-income respondents are more likely to have positive attitudes toward vertically farmed leafy vegetables. This could indicate that higher-income consumers are more willing to try novel foods produced using modern technologies. Meiselman et al. [40] showed that neophobia declines as income rises. The dummies for incomes of RUB 40,001–60,000 (“Income_46”) and of RUB 20,001–40,000 (“Income_24”) were not significant.

The gender dummy and dummies for age cohorts (30s, 40s, and over 50) were not statistically significant. These results suggest that neither gender nor age differences affected respondents’ attitudes.

### 3.3. High-Frequency Words

Table 4 shows the frequencies of words appearing in respondents’ answers to the open-ended question about their views. 

Although highly ranked words (e.g., “good,” “safe,” “tasty,” “fresh,” “healthy,” and “interesting”) sound positive, several words indicating negative meaning (e.g., “not_natural,” “less_nutritious,” “dangerous,” “not_ tasty,” and “bad”) were offered. Such assessments as “not_know” and “taste” bore neither positive nor negative connotations.

As expected, words having different connotations appeared in views on vertically farmed leafy vegetables. However, this simple tabulation of word frequency does not indicate how words are associated with degrees of favorability. Thus, we built a two-mode co-occurrence network using these words and respondents’ answers to favorability questions.

### 3.4. Two-Mode Co-Occurrence Network Map

**Figure 3:**
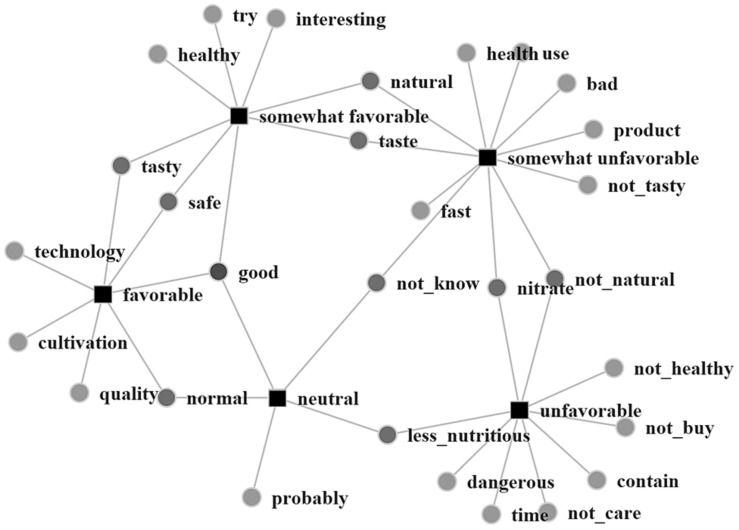
Two-mode co-occurrence network of frequent words and favorability.

Figure 3 maps a two-mode co-occurrence network with 40 edges in descending order of Jaccard coefficients. We set the number at 40, because too many or too few edges hampered the interpretation of the generated network.

Figure 3 can be interpreted as follows:

- Favorable: Words, such as “good,” “tasty,” “safe,” “quality,” and “technology”, appeared often in written comments from respondents who chose the “favorable” option. Their frequency indicates that some consumers had good impressions and that they believed leafy vegetables produced using the new technology were high in quality, safe, and tasty.
- Somewhat favorable: In comments by respondents who selected the “somewhat favorable” option, “good,” “tasty,” and “safe” again appeared frequently. However, words like “interesting,” “try,” “taste,” and “healthy” also appeared often. This finding suggests that respondents who had a somewhat positive feeling toward vertically farmed leafy vegetables also believed that they were safe and tasty. Furthermore, they were interested in trying them. Consumers in this group are expected to accept vertically farmed vegetables, depending on their quality, in particular their taste.
- Neutral: Respondents who reacted neutrally frequently mentioned “not_know,” “probably,” “good,” and “less_nutritious.” These words indicated no clear image of vertical farms and their produce. Because “not_know” is also connected to “somewhat unfavorable,” and “less_nutritious” is connected to “unfavorable,” the feelings of respondents who chose a neutral option presumably approximated those having negative emotions.
- Somewhat unfavorable: Relatively many words were linked to this option. “Not_know” again appeared frequently among respondents who selected the “somewhat unfavorable” option, indicating they also lacked a clear perception of vertical farms and their benefits. The occurrence of words (e.g., “product,” “not_natural,” “not_tasty,” “bad,” “health,” “use,” and “nitrate”) implies that respondents viewed vertically farmed leafy vegetables as unnatural, compared with vegetables grown outdoors. They tended to worry about the taste of the vegetables and the health effects of nitrates. Respondents’ use of the word “fast” shows that they believed vegetables grew quickly but unnaturally. The term, “fast growth,” is not necessarily a positive evaluation.
- Unfavorable: Words having negative meanings (e.g., “not_natural,” “not_healthy,” “less_nutritious,” “nitrate,” “dangerous,” “not_care,” and “not_buy”) appeared often among respondents who selected the “unfavorable” option. Members of this group were uneasy about consuming vegetables grown under artificial light because they believed that the vegetables were not natural, less nutritious, and even dangerous because of their nitrate contents.

## 4. Discussion

The results showed that consumers had heterogeneous attitudes toward vertically farmed vegetables, as in the case of other food technologies [10,17,18,19]. Although roughly half of the respondents had favorable attitudes, the remaining half expressed neutral or unfavorable attitudes, presumably because of misunderstandings or lack of knowledge. Consumers in the Central and Volga districts of western Russia or those with lower income were more likely to have a negative attitude toward vertically farmed vegetables. Such less favorably inclined respondents viewed them as unnatural, less nutritious, bad-tasting, and even dangerous, owing to nitrates. However, consumers in other districts or those having higher incomes were more likely to have a positive attitude and view the produce as good quality, safe, and tasty. These findings indicate that attitudes about vertically farmed vegetables were related to region of residence, income level, and opinions regarding “nutrients,” “safety,” and “taste.”

Implications for policymakers, vertical farmers, and marketers emerge from our findings. To dispel concerns and to make appeal to the quality of vertically farmed vegetables, it would be important to provide the public sufficient and accurate information about the vegetables’ nutrients, safety, and taste. Detailed but straightforward information about growing systems in vertical indoor farms would broaden public understanding of their necessity, features, and advantages.

It is important to inform consumers that vertically farmed vegetables can be as or more nutritious than vegetables grown outdoors. Amoozgar et al. [41] showed that the growth and nutritional value of lettuce could be enhanced by using LED light in indoor plant production facilities. Packaging or labeling that features nutrient information and charts or graphs comparing ingredients of vertically farmed and outdoor-grown vegetables would help consumers evaluate them. Over the long term, educational seminars and school sessions regarding such information could increase public understanding of their nutritional aspects.

Public disclosure of information about food safety management for vertical farms would augment consumer confidence. The perceived use of chemicals and the microbiological status of food products concern consumers. Again, education will enhance consumer understanding of the safety of vertical farming. Including information about producers on packaging might increase consumer confidence [17].

To address concerns about the taste of vertically farmed vegetables, promoters should provide consumers with opportunities to try them by offering trial samples at a store location or by holding tasting events. Supplying vertically farmed vegetables to school meal services could spark young people’s long-term interest in the technology.

Moreover, our findings provide regional and district marketing data that reflect consumer viability. Nonetheless, it is important to inform people in the Central and Volga districts of the merits of indoor farming in populous regions, including the carbon reductions related to fewer food miles and the benefits of high productivity in small spaces. This could be presented alongside information about nutritional value, safety, and taste.

The present study has some limitations. First, the inference that consumers see vertically farmed vegetables as less nutritious and tasty because they grow without sunlight and soil (i.e., artificial lighting and hydroponics) needs further investigation. Qualitative methods, such as focus groups or in-depth interviews could aid understanding of this issue. Second, the sample size was small relative to the target population and was skewed towards relatively younger age groups and populous regions regardless of the relatively high internet penetration rate in Russia. Increasing sample sizes or proportional quota sampling would be useful to overcome this problem. Finally, we did not test the empirical relationships among consumer perceptions, attitudes, and acceptance. Future empirical research with larger sample sizes is needed.

## 5. Conclusions

This study explored consumers’ attitudes and opinions toward vertically farmed vegetables in Russia. Using an ordered logit model and a two-mode co-occurrence network analysis, it examined how their attitudes related to their personal characteristics and opinions about the vegetables.

Our findings showed that consumers’ attitudes were heterogeneous and related to their region of residence, income, and opinions regarding nutrients, safety, and taste. Some expressed negative opinions about the naturalness and quality of the vegetables. It would be important to broaden public understanding of vertical indoor farming by providing sufficient information about its growing systems, food safety management, and the quality of its produce while comparing it to traditional farming.

There remains a need for further research to identify the reasons why consumers see vertically farmed vegetables as unnatural and less nutritious. Future research should also examine how information regarding vertical indoor farming affects consumers’ attitudes and purchase intentions toward its produce.

## Figures and Tables

**Table 1:** Sociodemographic characteristics of the survey participants (*n* = 289).

Characteristic		*n*	%	Characteristic		*n*	%
Gender	Female	149	51.6	Family size	1	13	4.5
	Male	140	48.4		2	58	20.1
Age (years)	20–29	70	24.2		3	110	38.1
	30–39	108	37.4		4	83	28.7
	40–49	71	24.6		More than 5	25	8.7
	50–59	31	10.7	Children under 12	Yes	164	56.7
	60–69	8	2.8		No	125	43.3
	70 or older	1	0.3	Monthly income (RUB)	Under 10,000	25	8.7
Region of residence	Central	111	38.4		10,001–20,000	36	12.5
	Northwest	40	13.8		20,001–30,000	59	20.4
	Southern	19	6.6		30,001–40,000	47	16.3
	North Caucasus	4	1.4		40,001–50,000	27	9.3
	Volga	58	20.1		50,001–60,000	26	9.0
	Urals	24	8.3		60,001–70,000	21	7.3
	Siberian	26	9.0		70,001–80,000	10	3.5
	Far East	7	2.4		Over 80,001	38	13.1

Source: Questionnaire survey.

**Table 2:** Respondents’ favorability toward vertically farmed leafy vegetables (*n* = 289).

Favorability	*n*	%
Favorable	59	19.5
Somewhat favorable	102	33.8
Neutral	57	18.9
Somewhat unfavorable	63	20.9
Unfavorable	21	6.9

Source: Questionnaire survey.

**Table 3:** Results of ordered logistic regression (*n* = 289).

	Full Model		Final Model (Backward Stepwise Selection)	
Variable	Coef.	Std. Err.	Coef.	Std. Err.
Gender	−0.034	0.235		
Age cohorts				
30s	−0.106	0.287		
40s	−0.212	0.322		
50s and over	−0.134	0.367		
Region of residence				
Central	−0.641**	0.276	−0.504 **	0.243
Northwest	−0.317	0.357		
Volga	−0.670 **	0.317	−0.547 *	0.288
Income groups				
Income_24	0.412	0.292		
Income_46	0.436	0.336		
Income_68	1.180 ***	0.423	0.845 **	0.352
Income_o8	1.132 ***	0.411	0.769 **	0.339
Threshold parameters				
Cut1	−2.735	0.446	−2.777	0.274
Cut2	−1.079	0.405	−1.129	0.193
Cut3	−0.020	0.403	−0.265	0.181
Cut4	1.448	0.412	1.372	0.201
Model summary				
Observations	289		289	
Pseudo R-squared	0.02		0.02	
Wald Chi-square	18.2 *		14.7 ***	
AIC	884.6		874.1	

Note: *** *p* < 0.01, ** *p* < 0.05, * *p* < 0.1. Cuts labeled as Cut 1, Cut 2, Cut 3, and Cut 4 are the estimated threshold parameters (cut-off points) of the latent variable. For example, “Cut 1” gives a cut-off point that differentiates “unfavorable = 1” and “somewhat unfavorable = 2.” For backward stepwise selection, variables are removed and added based on predefined significance threshold levels: the alpha-to-remove is set to 0.2 and the alpha-to-enter is set to 0.1. AIC: Akaike information criterion.

**Table 4:** Thirty most frequently occurring words (high-frequency words).

Rank	Word	Freq.	Percent	Rank	Word	Freq.	Percent
1	good	41	14.2		less_nutritious	18	6.2
2	not_know	32	11.1	17	fast	17	5.9
3	safe	29	10.0		dangerous	17	5.9
	tasty	29	10.0	19	nitrate	16	5.5
5	natural	28	9.7	20	not_tasty	15	5.2
6	quality	26	9.0		probably	15	5.2
7	not_natural	25	8.7	22	price	13	4.5
8	taste	24	8.3		technology	13	4.5
9	fresh	23	8.0		bad	13	4.5
10	healthy	22	7.6	25	not_care	12	4.2
11	product	21	7.3	26	clean	11	3.8
	interesting	21	7.3		innovation	11	3.8
13	use	20	6.9		cheap	11	3.8
14	normal	18	6.2		ecological	11	3.8
	cultivation	18	6.2	30	try	10	3.5

Source: Questionnaire survey.

## Data Availability

Data available on request.
